# The efficacy of Stereotactic body radiation therapy and the impact of systemic treatments in oligometastatic patients from prostate cancer

**DOI:** 10.1002/cam4.1707

**Published:** 2018-08-02

**Authors:** Ciro Franzese, Paolo Andrea Zucali, Lucia Di Brina, Giuseppe D'Agostino, Pierina Navarria, Davide Franceschini, Armando Santoro, Marta Scorsetti

**Affiliations:** ^1^ Radiotherapy and Radiosurgery Department Humanitas Clinical and Research Hospital Milan‐Rozzano Italy; ^2^ Medical Oncology and Hematology Department Humanitas Clinical and Research Hospital Milan‐Rozzano Italy; ^3^ Department of Biomedical Sciences Humanitas University Milan‐Rozzano Italy

**Keywords:** hormonal therapy, oligometastases, prostate cancer, stereotactic body radiation therapy, systemic treatment

## Abstract

**Background:**

Diagnoses of oligometastatic prostate cancer (PC) increased in the recent years thanks to the advancement in imaging and more effective systemic therapies. Here we evaluate the efficacy of Stereotactic Body Radiation Therapy (SBRT) in oligorecurrent and oligoprogressive PC.

**Methods:**

We included patients with a maximum of five metastases diagnosed in a maximum of two target organs. Concomitant treatment with hormonal therapies or chemotherapies was allowed. End points of the present study were the outcome in terms of Local control of treated metastases (LC), out‐field progression free survival, overall progression free survival (PFS), and overall survival.

**Results:**

We included in the analysis 64 patients treated on 90 metastases. Fifty (78.1%) patients were treated on lymph nodes, 2 (3.1%) patients simultaneously on lymph node and bone while 10 (15.7%) patients on bone only. Lung metastases were treated in 2 (3.1%) patients. Thirty‐seven (57.81%) were without androgen deprivation therapy when treated with SBRT. Median follow‐up was 15.2 months. Rates of LC at 6‐, 12‐, and 18‐ months were 94%, 88%, and 84%, respectively. Oligoprogressive patients compared to oligorecurrent (HR 9.10, *P* = 0.049) and prolongation of time from diagnosis of metastases to SBRT (HR 1.03, *P* = 0.047) were associated with worse LC. Median PFS was 6.6 months (range 1.1‐42.4). Castration resistant patients experienced worse PFS compared to castration sensitive group (HR 2.12, *P* = 0.021).

**Conclusions:**

Stereotactic body radiation therapy seems to be an effective treatment for metastases from PC. Prospective trials are necessary to better define selection of patients and to evaluate combination of SBRT and new systemic drugs in castration resistant patients.

## 1 INTRODUCTION

In 1995, Hellman and Weichselbaum coined the term “oligometastasis” as an intermediate state between localized tumor and widespread diffuse disease.^1^ Since that year several studies focused on the attempt to understand the benefit from local therapies on isolated metastases, with the conviction that oligometastatic patients are affected by a disease with a biology different from the classic concept of metastasis.^2^

Prostate cancer (PC) can metastasize to different sites. According to Harada et al^3^, in an autoptic series on 136 patients, 36% and 32% were identified with one and two bone lesions, respectively. The number of metastases that defines the oligometastatic state in PC is still unclear. A wide variability exists in the recent literature. Some studies limited the number of metastases to five^4^ and the majority of published trials included a maximum of three lesions.^5^, ^6^ Singh et al^7^ reported that a number of metastases limited to five lesions, developed during follow‐up after curative treatment of primary tumor, was significantly associated to better 5‐year survival (73% vs 43% of patients with more than five metastases). Diagnoses of oligometastatic PC increased in the recent years; possible causes of this increase include the advancement in imaging with new PET tracers, and the longer survival improved by more effective systemic therapies.^8^, ^9^, ^10^

According to the study of Sobol et al,^11^ among 2466 men underwent choline‐PET for suspected relapse, 134 (67%) were diagnosed with metastatic disease, (25% with axial or appendicular bone, and 75% with recurrences in the soft tissue]. De Bruycker et al analyzed prospectively 208 patients candidate to PET scan and showed that 153 patients (74%) had low‐volume recurrence, defined as isolated local recurrence or with ≤3 metastases (with or without local recurrence). Among 153, 119 had only metastatic recurrence.^12^ Historically, PC with any number of metastases was treated with systemic therapy, more commonly androgen deprivation therapy (ADT). Optimal management of oligometastatic patients is still to be defined. No real advantage from systemic treatments has been demonstrated in early recurrent PC. According to Duchesne et al,^13^ in the TOAD trial there was no improvement of survival from immediate ADT in PSA‐recurrent patients (*P* = 0.10). Gravis et al analyzed patient subgroups from the CHAARTED and GETUG‐AFU15 according to metastatic burden. While the analysis showed survival benefit from adding immediate docetaxel to ADT in high volume patients, no benefit was observed for low volume disease with HR of 1.03 (95% CI: 0.77; 1.38). The role of local approaches in oligometastatic PC patients has been investigated recently in few reports.^14^, ^15^, ^16^ Decaestaker et al^17^ treated 50 patients with 70 metastatic lesions from PC; the authors included hormone sensitive patients with up to three synchronous metastases in bones or lymph nodes. Local control was reached in 100% of sample and median PFS was 19 months with ¾ of patients recurring with a maximum of three metastases. Here we evaluate the efficacy of Stereotactic Body Radiation Therapy (SBRT) in oligorecurrent and oligoprogressive PC and the impact of systemic therapies on the behavior of disease.

## 2 MATERIALS AND METHODS

### 2.1 Study population

We included in this single institution analysis patients with histologically confirmed diagnosis of prostate adenocarcinoma, treated with surgery +‐ adjuvant/salvage radiotherapy (RT) or radical RT, which developed metachronous metastases during follow‐up from 2009 to 2016. All cases were presented to and approved by the multidisciplinary uro‐oncology team. The local ethics committee approved the analysis. Patients were candidate to SBRT if a maximum of three metastases with a maximum diameter of 5 cm were diagnosed in 1‐2 organs (eg bone and lymph node). Concomitant treatment with hormonal therapies or chemotherapies was allowed. All patients were staged with 11c‐choline PET or CT scan together with 99mTc‐bone scan. The study was conducted in accordance with Good Clinical Practice guidelines, the ethical principles of the Declaration of Helsinki and local regulations. Exclusion criteria were patients with diagnosis of synchronous metastases.

### 2.2 Techniques of radiotherapy

The clinical target volume (CTV) was equal to gross tumor volume (GTV) and was delineated on simulation CT imaging, coregistered with MRI scan or PET scan when available. In‐vein contrast for CT was used in case of treatment of lymph node metastases. In case of disease located into organ subject to internal movement (such as lung), patients were simulated with 4D‐CT scan. All patients were positioned supine, with a thermoplastic mask, both for abdomen and pelvis. An isotropic margin of 5‐10 mm, depending on disease site and dimensions, was added to CTV to obtain the planning target volume (PTV). In case dose constraints for organs at risk were not met, dose to PTV was deescalated. All patients were treated with Volumetric Modulated Arc Therapy technique. The patient's position was evaluated daily with Cone‐beam CT imaging before each treatment session. Patients treated with systemic therapy were submitted to SBRT for comparison on new isolated sites of disease (Oligorecurrence) or for progression of few sites while the remaining were controlled by systemic therapy (Oligoprogression).

### 2.3 Response assessement

First evaluation was planned 3 months after the end of the SBRT and then every 3 months for the first year and every 6 months from the second to the fifth year. Clinical evaluation and PSA values were obtained for every follow‐up visit. Diagnostic imaging (CT, Choline‐PET or MRI scan) was planned at physician choice. In general reassessments with imaging were planned in case of three rising PSA values after response or in case of PSA rise above the pre‐SBRT value or in case of new potentially disease related symptoms. Tumor response was classified according to European Organization for Research and Treatment of Cancer Response Evaluation Criteria In Solid Tumors (EORTC‐RECIST) criteria version 1.16. PET Response Criteria in Solid Tumors (PERCIST)^18^ were used to evaluate metabolic response in patients who underwent PET scan for restaging. Globally castration resistant patients’ were evaluated after treatment according to Prostate Cancer Clinical Trials Working Group 3 (PCWG3).^19^

### 2.4 Statistical analysis

Data were collected and analyzed retrospectively. End points of the present study included the outcome in terms of local control of treated metastases (LC), out‐field progression free survival (OF‐PFS), progression free survival (PFS), and overall survival (OS). Local control was analyzed at patient's level and defined as the time from the beginning of SBRT to the in‐field progression of treated metastases or last follow‐up. Out‐field progression free survival was defined as the time from the SBRT to the onset of new metastases. Additionally PFS was defined as the time from SBRT to the evidence of in‐field or out‐field progression or increase of PSA. Overall survival was calculated from the SBRT to either death or last follow‐up. Univariate analysis was performed with the log‐rank test, and Cox proportional hazards regression was used to estimate hazard ratios (HR). Multivariable Cox regression analysis was performed to evaluate the association between clinical factors and survival, with a significance level of *P* < 0.05. Statistical calculations were performed using STATA, version 14 (StataCorp 2015, College station, Texas: StataCorp LP.).

## 3 RESULTS

A total of 64 PC patients with 90 metastases were treated with SBRT. Patients’ characteristics are summarized in Table 1. Median age was 71.8 years (range 51.3‐82.9) and median PSA at first diagnosis (iPSA) was 9.95 ng/mL (range 3.7‐146). Forty‐eight (75%) patients were treated on primary tumor with radical surgery while 5 (7.81%) patients underwent radical RT. The remaining patients underwent ADT (7.81%) or high intensity focused ultrasound (9.38%) as first treatment. Among patients treated with surgery, 35 patients (54.6%) had adjuvant or salvage RT. At first biochemical relapse median PSA was 1.3 ng/mL (range 0.03‐146) and median time from diagnosis primary tumor to detection of metastases was 34.8 months (range 0‐194.3). Median time from detection of metastases to SBRT was 1.85 months (range 0.23‐58.5). Median PSA before SBRT was 2.16 ng/mL (range 0.03‐28.62). The majority of patient performed PET before the RT treatment while only two patients had CT scan for diagnosis of metastatic disease. Patients were more commonly treated on abdominal or pelvic lymph nodes only (50 patients, 78.1%). Two (3.1%) patients were treated simultaneously on lymph node and bone metastases while 10 (15.7%) patients on bone metastases only. Lung metastases were treated in 2 (3.1%) patients. According to American Joint Committee on Cancer (AJCC) TNM Staging System For Prostate Cancer (8th ed., 2017), patients were classified as N1 (35; 54.7%) if only regional lymph node were involved, M1a (15; 23.4%) if nonregional lymph nodes metastases were detected, M1b (12; 18.7%) if treated for bone metastases. Visceral metastases were classified as M1c (2; 3.1%). Globally 45.3% (29) patients were classified as M1. Forty‐one (64.1%) patients were treated with SBRT on one single metastasis while 20 (31.2%) and 3 (4.7%) patients on two and three metastases, respectively. Only two patients had other sites of disease not treated with SBRT but controlled by systemic therapy.

**Table 1:** Patient's characteristics

	N (%)
Age median (range)	71.8 (52.9‐82.9)
≤65	9 (14%)
>65	55 (86%)
PS	
0	39 (60.9%)
1	19 (29.7%)
2	6 (9.4%)
Gleason score	
≤8	47 (73.4%)
>8	17 (26.6%)
PSA at diagnosis, median ng/mL (range)	9.95 (3.7‐46)
NCCN Risk group	
Low risk	2 (3.1%)
Intermediate risk	35 (54.7%)
High risk	27 (42.2%)
Initial treatment	
Surgery	13 (20.3%)
Surgery + Radiotherapy	35 (54.7%)
HIFU	6 (9.4%)
ADT	5 (7.8%)
Radiotherapy	5 (7.8%)
Time to biochemical relapse, median months (range)	32.8 (0‐169.6)
Time to metastases, median months (range)	34.8 (0‐194)
Number of treated metastases	
1	41 (64%)
2	20 (31.2%)
3	3 (4.7%)
Site of metastases	
Lymph node	52 (81.2%)
Bone	10 (15.6%)
Lung	2 (3.1%)
American joint committee on cancer TNM staging	
N1	35 (54.7%)
M1a	15 (23.4%)
M1b	12 (18.7%)
M1c	2 (3.1%)
Oligorecurrence vs oligoprogressive disease	
Oligorecurrence	62 (96.9%)
Oligoprogression	2 (3.1%)

Radiotherapy was delivered with a median dose of 42 Gy (range 18‐60) in 2‐8 fractions. Median dose per fraction was 7.5 Gy (range 5‐12). Thirty‐seven (57.81%) were without ADT when treated with SBRT. Twenty‐seven patients were treated with ADT when submitted to radiation. Nineteen (29.7%) patients were classified as castration‐resistant when treated with SBRT on metastases. Treatment's characteristics are summarized in Table 2.

**Table 2:** Treatments’ characteristics

	N (%)
Systemic therapy during RT	
No	37 (57.8%)
Yes	27 (42.2%)
Previous ADT	
No	16 (25%)
Yes	48 (75%)
Previous chemotherapy	
No	51 (79.7%)
Yes	13 (20.3%)
Lines of systemic therapies	
0	15 (23.4%)
1	25 (39%)
2	12 (18.7%)
3	12 (18.7%)
Castration sensitive vs resistant disease	
Sensitive	45 (70.3%)
Resistant	19 (29.7%)
Time from M+ to SBRT, median months (range)	1.8 (0.2‐58.5)
PSA before SBRT, median ng/mL (range)	2.16 (0.03‐28.62)
SBRT total dose, median Gy (range)	42 (18‐60)
5 Gy x 5	2
5 Gy x 6	3
6 Gy x 5	9
6 Gy x 6	7
7.5 Gy x 6	15
8 Gy x 4	1
9 Gy x 2	1
10 Gy x 4	6
10.5 Gy x 4	5
12 Gy x 4	15
BED3, median Gy (range)	157 (66.6‐240)
≤100	15 (23.4%)
>100	49 (76.6%)

Median follow‐up time was 15.2 months (range 3‐101.4). Best radiologic response after SBRT was classified as complete response in 41 (64.1%) patients, partial response in 10 (15.6%) patients, and stable disease in 2 (3.1%) patients. Eight patients didn't performed radiological examination for evaluation of response and were evaluated only with PSA value. Overall median value of nadir PSA (nPSA) reached after SBRT was 1.64 ng/mL (range 0‐45.05). Median nPSA in patients without progression after RT was 0.39 ng/mL (range 0.01‐5.8). Biochemical response was observed in 25 patients.

**Figure 1:**
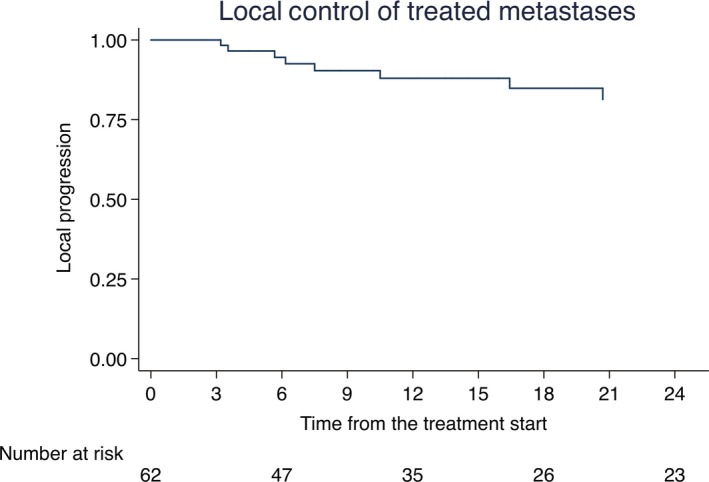
Kaplan‐Meier of Local control of treated metastases Local control of treated metastases at 6‐, 12‐, and 18‐mo was 94% (95% CI: 0.56‐0.80), 88% (95% CI: 0.25‐0.51), and 84% (95% CI: 0.13‐0.38), respectively

Local control of treated metastases at 6‐, 12‐, and 18‐ months was 94% (95% CI: 0.56‐0.80), 88% (95% CI: 0.25‐0.51), and 84% (95% CI: 0.13‐0.38), respectively. Median time to in‐field progression was 14.1 months (range 2.5‐101.4). Figure 1 illustrates LC Kaplan‐Meier analysis. At univariate analysis oligoprogressive patients compared to oligorecurrent (HR 9.10, 95% CI: 1.00‐82.32, *P* = 0.049) and prolongation of time (per unit of month) from the diagnosis of metastases to SBRT (HR 1.03, 95% CI: 1.00‐1.07, *P* = 0.047) were associated with a worse in‐field control. At multivariable analysis none of the analyzed factors was statistically significant even if borderline, as summarized in Table 3.

**Table 3:** Univariate and multivariate analysis for Local control

	Univariate			Multivariable		
	HR	95% CI	*P* value	HR	95% CI	*P* value
Age	1.05	0.93‐1.20	0.373	‐	‐	‐
PS	2.26	0.91‐5.58	0.076	‐	‐	‐
NCCN class risk	0.62	0 0.18‐2.09	0.450	‐	‐	‐
Time to metastases	1.00	0.98‐1.01	0.787	‐	‐	‐
Time to biochemical relapse	1.00	0.98‐1.01	0.656	‐	‐	‐
Site of metastases	2.13	0.70‐6.47	0.182	‐	‐	‐
Number of treated metastases	0.51	0.11‐2.24	0.374	‐	‐	‐
Oligorecurrence vs oligoprogressive	9.10	1.00‐82.32	0.049	8.71	0.88‐85.8	0.064
Time to SBRT	1.03	1.00‐1.07	0.047	1.03	0.99‐1.07	0.055
PSA before SBRT	1.04	0.95‐1.12	0.339	‐	‐	‐
Systemic treatment during SBRT	1.37	0.34‐5.50	0.654	‐	‐	‐
Lines of systemic treatments	0.98	0.52‐1.86	0.966	‐	‐	‐
Castration sensitive vs resistant	0.72	0.14‐3.57	0.688	‐	‐	‐
ADT before SBRT	1.48	0.35‐6.21	0.588	‐	‐	‐

Thirty‐eight (59.3%) patients were diagnosed with out‐field distant metastases with a median time of 8.1 months (range 1.6‐46.4). In particular, out‐filed progression was observed in 25 castration sensitive patients and 13 castration resistant patients. Rates OF‐PFS were 78% (95% CI: 65%‐86%), 52% (95% CI: 38%‐65%), and 37% (95% CI: 23%‐51%) at 6‐, 12‐, and 18‐ months, respectively.

**Figure 2:**
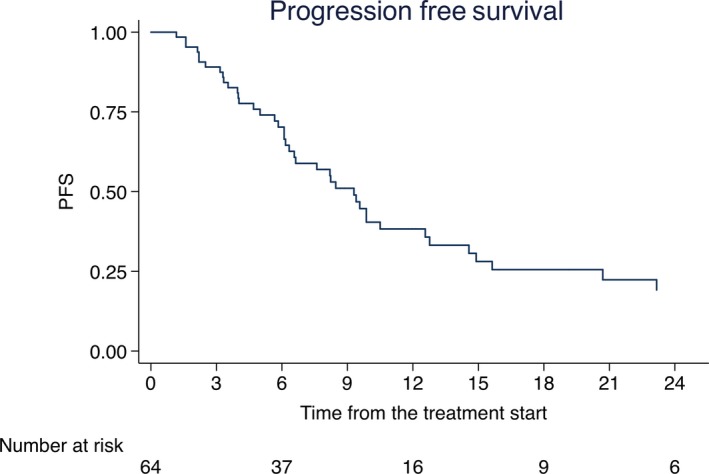
Kaplan‐Meier of Progression Free Survival. Rates of Progression Free Survival at 6‐, 12‐, and 18‐mo were 70% (95% CI: 56%‐80%), 38% (95% CI: 25%‐51%), and 25% (95% CI: 13%‐38%)

**Figure 3:**
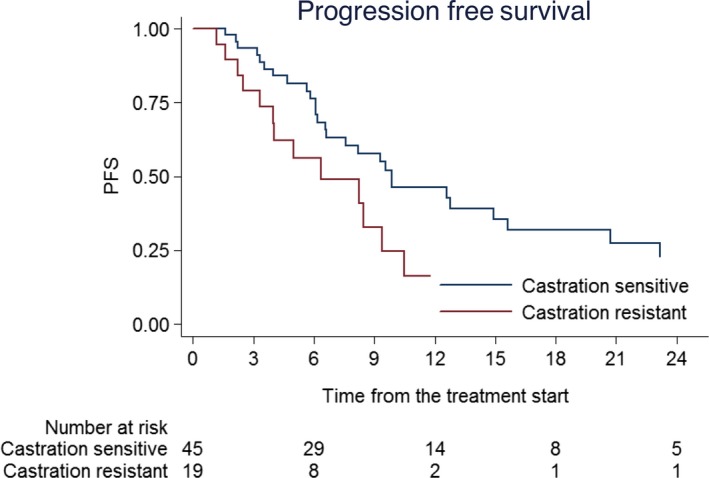
Kaplan‐Meier of Progression Free Survival according to sensitivity to hormonal therapy. Progression Free Survival at 6‐, 12‐, and 18‐mo were 76% (95% CI: 60%‐86%), 46% (29%‐61%), and 32% (17%‐48%), respectively, for castration sensitive, and 56% (30%‐75%), 16% (2%‐40%), and 8% (0%‐30%) for castration resistant subgroup

Globally 44 (68.7%) patients had in‐field or out‐field progression of disease after SBRT with a median PFS of 6.6 months (range 1.1‐42.4). Rates of PFS at 6‐, 12‐, and 18‐ months were 70% (95% CI: 56%‐80%), 38% (95% CI: 25%‐51%), and 25% (95% CI: 13%‐38%) as in Figure 2. Analysis of correlation between risk factors and PFS is summarized in Table 4. Castration resistant patients experienced a worse PFS compared to castration sensitive group (HR 2.12, 95% CI: 1.11‐4.03; *P* = 0.021). Rates of PFS at 6‐, 12‐, and 18‐ months were 76% (95% CI: 60%‐86%), 46% (95% CI: 29%‐61%), and 32% (95% CI: 17%‐48%), respectively for castration sensitive, and 56% (95% CI: 30%‐75%), 16% (95% CI: 2%‐40%), and 8% (95% CI: 0‐30%) for castration resistant subgroup (Figure 3). Median PFS for castration resistant patients was 6.3 months vs 9.8 months for castration sensitive group. All patients were alive at the moment of the analysis.

**Table 4:** Univariate and multivariate analysis for PFS

	Univariate			Multivariable		
	HR	95% CI	*P* value	HR	95% CI	*P* value
Age	0.97	0.93‐1.02	0.354	‐	‐	‐
PS	0.82	0.51‐1.33	0.434	‐	‐	‐
NCCN class risk	0.70	0.40‐1.21	0.205	‐	‐	‐
Time to metastases	0.99	0.99‐1.00	0.402	‐	‐	‐
Time to biochemical relapse	0.99	0.98‐1.00	0.253	‐	‐	‐
Site of metastases	1.01	0.51‐2.00	0.955	‐	‐	‐
Number of treated metastases	1.59	0.94‐2.70	0.081	‐	‐	‐
Oligorecurrence vs oligoprogressive	3.03	0.39‐23.05	0.283	‐	‐	‐
Time to SBRT	1.00	0.98‐1.02	0.526	‐	‐	‐
PSA before SBRT	1.02	0.98‐1.06	0.297	‐	‐	‐
Systemic treatment during SBRT	1.79	0.97‐3.29	0.061	‐	‐	‐
Lines of systemic treatments	1.37	0.84‐2.22	0.199	‐	‐	‐
Castration sensitive vs resistant	2.12	1.11‐4.03	0.021	2.12	1.11‐4.03	0.021
ADT before SBRT	1.38	0.75‐2.55	0.295	‐	‐	‐

## 4 DISCUSSION

Here we assessed the impact of SBRT in oligometastatic PC patients. We evaluated 64 patients with 90 metastases undergoing high dose ablative RT. The results showed a LC rate of 88% at 1 year. High local control rates are reported in literature in this setting. Ahmed et al^16^ and Habl et al,^20^ observed a similar 100% of local control when treating 17 and 15 patients respectively. Muacevic et al.^14^ treated 64 bone metastases with single fraction SBRT reaching a rate of local control of 95.5%. Recently, Ost et al published a prospective randomized study comparing surveillance with metastasis‐directed therapy for oligometastatic PC. The analysis showed that the median ADT‐free survival was 13 months for the surveillance and 21 months for the treatment group (*P* = 0.11).^21^

Control of limited burden of disease could be a relevant point in PC patients, in whom the number of metastases are considered an important prognostic factor.^22^, ^23^ Globally we observed control of treated disease for a median time of 14.1 months; the time was prolonged to 15.9 months in patients who didn't experienced in‐field progression. Oligoprogressive patients seem to be characterized by a worse LC, however this data could be affected by imbalance in the sample, being only two patients in oligoprogression. Time to SBRT correlated to the response to local treatment, indeed the effect is quite light with an increased risk of 3%. Main question is the identification of patients actually oligometastatic who can benefit from SBRT as local approach. In our study, PFS value at 18 months was 25%, with high percentiles reaching 38%. Data are in line with the one reported for other solid tumor, about 35% of PFS at 2 years.^24^

The majority of our patients relapse out‐field after 18 months (63%) with about 50% after the first year. The addition of systemic treatment to SBRT seems to not affect the out‐field progression in our sample. These results confirm the need to better understand the selection of patients who can benefit from SBRT or the advantage to associate systemic treatments to radiotherapy. Our results are consistent with those published by other authors. For example Habl et al^20^ reported that ADT had no impact on PFS, time to initiation of ADT, or to intensification of systemic therapy. Also Jereczek‐fossa et al^25^ demonstrated that time to progression after SBRT for nodal metastases was similar whether androgen deprivation was added or not (11 vs 10 months).

According to our analysis, PFS seems to be worse in castration resistant PC. One year PFS rates were 46% (29%‐61%) for ADT sensitive patients vs 16% (2%‐40%) for castration resistant patients. For several years the only available treatment for castration resistant PC was chemotherapy, in particular docetaxel. Only in the recent decade the introduction of new generation hormonal therapies improved outcome of this subset of patients. The efficacy of abiraterone and enzalutamide has been proved by several prospective randomized trials, both in pre and postchemotherapy setting.^26^, ^27^, ^28^, ^29^, ^30^, ^31^ The AFFIRM trial^30^ that investigate the use of Enzalutamide postdocetaxel demonstrated a radiographic PFS (rPFS) of 8.3 months against 5.4 months of placebo. The COU‐AA‐301^31^ explored the efficacy of abiraterone in the same setting and demonstrated a rPFS 5.6 (2.0 in placebo). Higher rates of PFS are reported by trials of prechemotherapy setting (rPFS of 16.5 months of COU‐AA‐302 trial^27^ and median not reached in PREVAIL trial^28^). The castration resistant patients treated in our study reached a median rPFS of 8.4 months. Patients diagnosed as castration resistant when treated with SBRT are more likely to have subclinical disease that becomes evident during posttreatment follow‐up. However the role of local treatment in metastatic castration resistant prostate cancer patients has not been investigated, including the addition of new generation hormonal therapy to SBRT.

The present study is limited by several issues, including its retrospective nature and the short duration of follow‐up, due to which we were not able to analyze the impact on overall survival.

## 5 CONCLUSIONS

Our results confirm that SBRT in an effective treatment for oligorecurrent and oligoprogressive metastases from PC. Ablative RT could be beneficial in the oligometastatic setting where the treated metastases can sometimes be the only burden of disease. However studies of association of SBRT with systemic therapies, including new generation hormonal therapy, are necessary. The combination of new hormonal therapy and SBRT could potentially give the best results in selected patients.

## CONFLICT OF INTEREST

There are no conflict of interest disclosures from any authors.
